# The Pivotal Role of Thiamine Supplementation in Counteracting Cardiometabolic Dysfunctions Associated with Thiamine Deficiency

**DOI:** 10.3390/ijms26073090

**Published:** 2025-03-27

**Authors:** Giovanna Ritorto, Sara Ussia, Rocco Mollace, Maria Serra, Annamaria Tavernese, Ernesto Palma, Carolina Muscoli, Vincenzo Mollace, Roberta Macrì

**Affiliations:** 1 Pharmacology Laboratory, Institute of Research for Food Safety and Health IRC-FSH, Department of Health Sciences, University Magna Graecia of Catanzaro, 88100 Catanzaro, Italy; giovanna.ritorto@studenti.unicz.it (G.R.); saraussia1598@gmail.com (S.U.); muscoli@unicz.it (C.M.); mollace@libero.it (V.M.); robertamacri85@gmail.com (R.M.); 2 Department of Experimental Medicine, University “Tor Vergata” of Rome, 00133 Rome, Italy; 3 Department of Medicine and Surgery, University Campus Bio-Medico of Rome, 00128 Rome, Italy; an.tavernese@gmail.com; 4 Veterinary Pharmacology Laboratory, Institute of Research for Food Safety and Health IRC-FSH, Department of Health Sciences, University Magna Graecia of Catanzaro, 88100 Catanzaro, Italy; palma@unicz.it; 5 Renato Dulbecco Institute, Lamezia Terme, 88046 Catanzaro, Italy

**Keywords:** thiamine imbalance, thiamine supplementation, cardiovascular disease, atherosclerosis, diabetes mellitus, metabolic dysfunction

## Abstract

The isolation, structural elucidation, and synthesis of pure thiamin were achieved in 1936, marking a milestone in vitamin research. As an organic compound soluble in water, thiamin is essential for carbohydrate metabolism in plants and animals, and in its active form—as part of the thiamin pyrophosphate coenzyme—performs these functions. Thereby, thiamin represents an essential vitamin to human health and is involved in several pathways that regulate several pathophysiological mechanisms. Cardiovascular disease is significantly impacted by thiamine imbalance and its supplementation offers substantial improvements to the associated conditions. In this comprehensive review, we aimed to examine the dual role of thiamine deficiency and accumulation, focusing on an analysis of the causes of thiamine deficiency. We detailed the effects of thiamine deficiency on metabolism and on cardiovascular risk and heart failure, explaining the molecular mechanisms involved in metabolic dysfunction, and highlighting the role of B1 vitamin supplementation in diabetes mellitus management and atherosclerosis development and progression. Indeed, B1 supplementation counteracts oxidative stress and inflammation, significantly ameliorating glycemic and lipemic profiles. Additionally, we reported the beneficial effects of thiamine in counteracting cardiotoxicity induced by cancer therapy. Although preclinical data strongly support the benefits of thiamine, clinical trial findings are in contrast and contradictory, hampered by limitations such as small sample sizes and inadequate follow-up. Further research is needed to investigate thiamine’s potential benefits, overcoming current study limitations and evaluating its use as a supplemental therapy alongside standard treatments in different high-cardiovascular-risk conditions.

## 1. Introduction

Hydrosoluble vitamin B1—also known as thiamine or aneurin—is essential to human biological pathways, growth, neurological and cognitive functions, development, and heart muscle contraction [1,2].

Thiamine is identified by its chemical name of 3-[(4-amino-2-methyl-5-pyrimidinyl) methyl]-5-(2-hydroxyethyl)-4-methylthiazolium [3].

A short methylene bridge connects the thiamine molecule, which is compounded by two heterocyclic rings forming a pyrimidine (4-amino-2-methylpyrimidine) and thiazole (4-methyl-5-(2-hydroxyethyl)-thiazolium) system [4].

Thiamine is available in a cationic (T+) status at physiological pH; however, in the intestinal lumen, before absorption, dietary thiamine is converted to free thiamine by intestinal phosphatases.

The highest recommended daily allowance (RDA) of thiamine is 1.3 mg/day, and it is influenced by sex and age group [5].

In particular, according to the 1980 Committee on Dietary Allowance, Food, and Nutrition Board, the estimated average requirement (EAR) for thiamine was established at 1 mg/day for adult women and 1.2 mg/day for adult men [6].

Despite being unhealthy, processed foods can easily meet—and may surpass—the recommended daily allowance of thiamine. The recommended daily thiamine intake is readily achieved through one serving of breakfast cereal, several slices of bread, or similar fortified foods [7].

Nevertheless, almost 28% of the population does not meet the recommended daily intake of this vitamin [8].

Though stable at acidic pH, free thiamine degrades in UV or gamma radiation and extreme temperatures. Oxidation of thiamine under alkaline conditions yields thio-chrome, a fluorescent compound useful for determining vitamin concentrations in food and medicine [9].

Conversely, thiamine deficiency can be caused by high temperature, alkaline pH, and the intake of coffee, tea, and other tannin-rich beverages [10,11].

The absorption of thiamine is significantly influenced by certain conditions, including alcohol consumption, anti-thiamine diet factors, food processing methods, and levels of protein and folate nutrition.

Many individuals cannot produce thiamine and thus have to include it in their diets. Low energy expenditure, metabolic requirements, genetic changes, and dietary thiamine levels in their natural food contributed to an animal’s lack of thiamine production [12]. The ability to biosynthesize thiamine is restricted to plants, fungi, microorganisms, and intestinal bacteria and dietary sources of thiamine are especially rich in whole grains, yeasts, cereals, and other products of plant origin, meats (pork), fish (tuna, salmon, catfish, and trout), legumes, nuts, and fortified foods [7,13].

Food processing, especially fortification, has proven to be an effective strategy: it has notably reduced thiamine-related deaths in The Philippines, Asia’s first nation to successfully fortify with thiamine; in addition, while fortification programs show success in specific groups, thiamine supplements offer another way to treat and prevent thiamine deficiency for individuals and communities [14].

Thus, providing critically ill patients with thiamine could boost their recovery prospects [15].

Dietary thiamine absorption mainly occurs in the jejunum, utilizing facilitative transport for delivery to the liver and incorporation into red blood cells (RBCs) [11].

TPP is approximately 80% of thiamine in these cells, while plasma levels are very depleted. Otherwise, the heart exhibits the highest level of thiamine (0.28–0.79 mg/100 g) [16].

Active or passive transport depends on the intake level. Active thiamin absorption in the intestine increases with lumen concentration, while passive absorption increases as concentration decreases [10].

The solute carrier family of genes contains two primary thiamine transporters: human thiamine transporter-1 (hTHTR-1), codified by solute carrier family 19 member 2 (SLC19A2) and human thiamine transporter-2 (hTHTR-2), encoded by solute carrier family 19 member 3 (SLC19A3). Thiamine is delivered from the intestine to the portal circulation through the basolateral membrane and then enters erythrocytes by facilitating hTHTR-1 [16].

A variety of other transporters such as solute carrier family 22 member 1 (SLC22A1) organic cationic transporter 1 (OCT1), solute carrier family 25 member 19 (SLC25A19) mitochondrial thiamin pyrophosphate transporter 1 (MTPP-1), solute carrier family 19 member 1 (SLC19A1) (folate transporter), solute carrier family 35 member F3 (SLC35F3), and solute carrier family 44 member 4 (SLC44A4) human thiamine pyrophosphate transporter (hTPPT/TPPT-1) are also involved in thiamine absorption [7].

Changes in human thiamine concentrations result from alterations in transporter function, which can be achieved through epigenetic mechanisms or common drugs [7].

Four identified genetic defects cause impaired thiamine transport and metabolism [7,8]; specifically, the outcomes of SLC19A2 dysfunction due to genetic mutation may include megaloblastic anemia (TRMA), sensory-neural hearing loss (sensorineural deafness), hyperglycemia, and diabetes mellitus [16].

**Figure 1:**
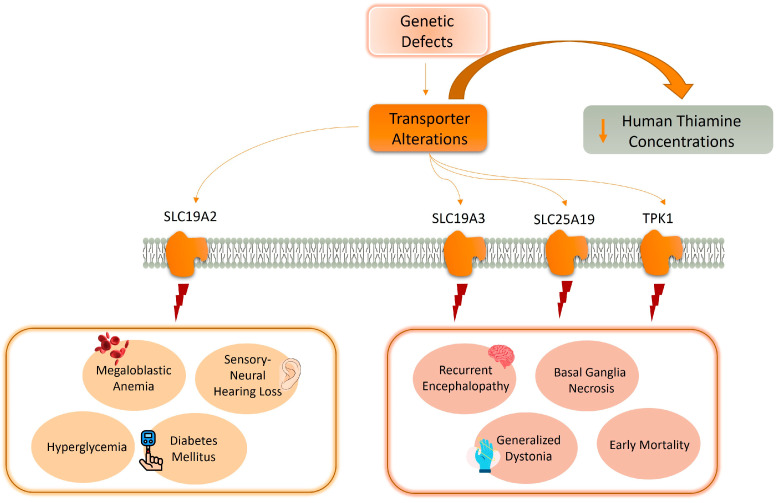
Several genetic defects cause alterations in the thiamine transporters, thus affecting its concentrations in the human body. Specifically, the outcomes of SLC19A2 dysfunction due to genetic mutation may include megaloblastic anemia (TRMA), sensory-neural hearing loss (sensorineural deafness), hyperglycemia, and diabetes mellitus. Furthermore, disorders associated with SLC19A3, SLC25A19, and TPK1 may result in recurrent encephalopathy, basal ganglia necrosis, generalized dystonia, and early mortality. Solute carrier family 19 member 2 (SLC19A2); solute carrier family 19 member 3 (SLC19A3); solute carrier family 25 member 19 (SLC25A19); thiamin pyrophosphokinase 1 (TPK1).

Furthermore, disorders associated with SLC19A3, SLC25A19, and thiamin pyrophosphokinase 1 (TPK1) may result in recurrent encephalopathy, basal ganglia necrosis, generalized dystonia, severe disability, and early mortality [8]; indeed, SLC25A19 pathogenic variants severely reduce mitochondrial TPP, causing Amish lethal microcephaly and thiamine metabolism dysfunction syndrome 4 (Figure 1) [8].

The biological half-life of thiamine ranges from 9 to 18 days and, according to metabolism and deactivation, thiamine is metabolized by two types of thiaminases: type I and type II. These enzymes cleave thiamine into its pyrimidine and thiazole components. Thiaminase I can be found in fish, ferns, some bacteria, and crustaceans, while Thiaminase II is detected in a few bacteria. Thiaminases are typically susceptible to extreme temperatures and can be destroyed by cooking. However, consuming too many thiaminase-containing foods or preparing them incorrectly can cause a thiamine deficiency [17].

Depending on individual thiamine levels, excess free thiamine is either excreted by distal nephrons or reabsorbed, with thiamine loss directly correlating to renal clearance; however, thiamine metabolites are unable to be reabsorbed by the kidney [7,11].

**Figure 2:**
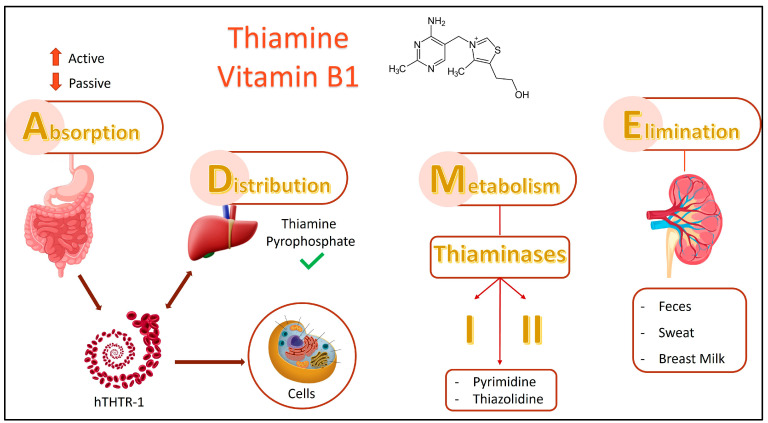
Thiamine absorption in the diet is centered in the jejunum of the small intestine; consequently, facilitative transport is employed to transfer it to the liver and introduce it into red blood cells. Active or passive transport determines the level of intake. Active absorption occurs when thiamin levels increase in the intestinal lumen, while passive absorption occurs when they decrease. Through the basolateral membrane, thiamine is transported from the intestine to the portal circulation, where it is then transported into erythrocytes with the assistance of hTHTR-1. The metabolism and deactivation of thiamine involves the metabolization of two types of thiaminases: type I and type II. These enzymes cleave thiamine into its pyrimidine and thiazole components. Excess free thiamine can either be excreted or reabsorbed by the body through distal nephrons. Additionally, thiamine is excreted in feces, principally due to bacterial thiamine, as well as sweat and breast milk. Human thiamine transporter-1 (hTHTR-1).

Interestingly, the excretion of metabolites remains unchanged despite thiamine deficiency. The glomerulus filters thiamine and other small solutes, and the proximal tubule metabolizes this filtered thiamine; however, thiamine within blood cells or bound to proteins is unfilterable. Within the tubule, phosphorylated thiamine—mostly as TMP—is converted to its unphosphorylated form (Figure 2) [17].

Renal tubular cell reuptake of thiamine from urine occurs at the basolateral membrane via thiamine transporter-1 (hTHTR1) and thiamine transporter-2 (hTHTR2), and at the brush border membrane via OCT1; THTR1 and organic cation transporter 2/3 (OCT2/3) contribute to this process on the basolateral membrane [9].

The use of loop diuretics such as furosemide in treatments leads to a notable reduction in thiamine, resulting in a twofold increase in basal levels [16]; additionally, bacterial thiamine, sweat, and breast milk primarily cause thiamine excretion in feces [9].

Due to the small accumulation of thiamine in tissues, the body’s supplies can be depleted within 2–3 weeks. Therefore, consuming a sufficient amount of thiamine regularly is necessary to satisfy the body’s continuous metabolic requirements [6].

Thiamine monophosphate (TMP), diphosphate, and triphosphate are the different forms of thiamine found in the human body [18].

**Figure 3:**
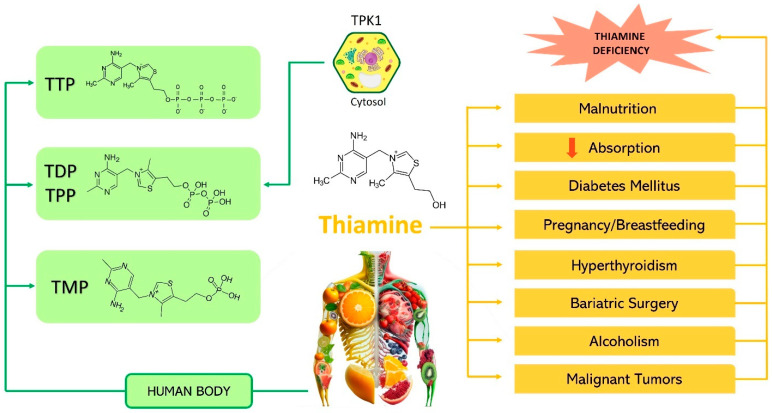
Thiamine is available in different forms in the human body, including thiamine monophosphate, thiamine diphosphate, and thiamine triphosphate. TPK1 phosphorylates the increased thiamine concentration in the cytosol to form thiamine diphosphate (TDP), also commonly referred to as thiamine pyrophosphate (TPP), the biologically active form of thiamine. The promotion of thiamine deficiency is immediate when combined with malnutrition, decreased gastro-intestinal absorption due to disease or surgery, chronic alcoholism, malignant tumors, diabetes mellitus (DM) with increased urinary excretion of thiamine, in pregnant or lactating women, and patients with hyperthyroidism, which may be linked to an increased metabolic requirement. Thiamine monophosphate (TMP); thiamine diphosphate (TDP); thiamine pyrophosphate (TPP); thiamine triphosphate (TTP); thiamine pyrophosphokinase (TPK1).

In humans, the homodimeric enzyme TPK1 is found in all cell types, predominantly in the small intestine, testes, and kidneys (Figure 3) [3], and TPK1-mediated thiamine phosphorylation is critical for efficient thiamine transport.

Cytosolic TPK1 phosphorylates the increased concentration of absorbed thiamine, producing thiamine diphosphate (ThDP)—also known as thiamine pyrophosphate (TPP)—the active form of the vitamin [3,4,8].

Furthermore, the dephosphorylation of adenosine triphosphothiamine, either by proteolytic enzymes or via thiamine triphosphate, yields thiamine diphosphate (ThDP) and releases coenzymes [3].

**Figure 4:**
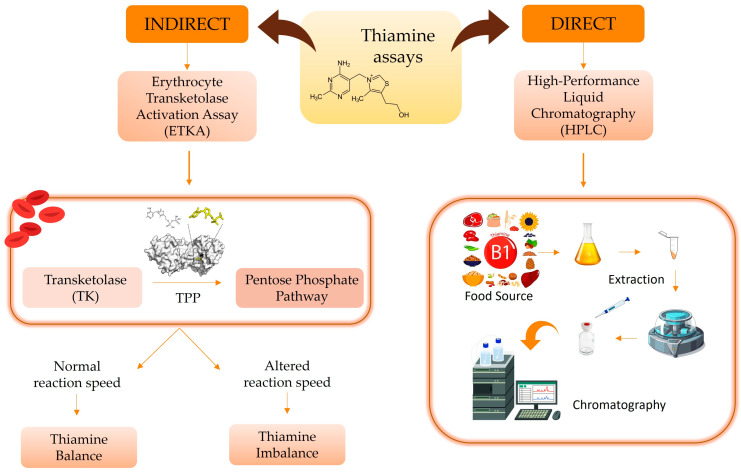
Several approaches can be used to assess thiamine levels: indirect measurement using erythrocyte transketolase activation assay (ETKA); and direct measurement through high-performance liquid chromatography (HPLC). The erythrocyte transketolase activation assay is based on the evaluation of the reaction speed of the transketolase that uses TPP as a cofactor. If the reaction rate is in the normal range, thiamine levels are balanced; on the contrary, if the speed is high, thiamine levels will be unbalanced. High-performance liquid chromatography is performed following extraction processes. Erythrocyte transketolase activation assay (ETKA); high-performance liquid chromatography (HPLC); transketolase (TK); thiamine pyrophosphate (TPP).

In assessing thiamine efficacy, biologically active thiamine (TPP) is considered the gold standard [18], and several approaches predict thiamine levels: indirect measurement using erythrocyte transketolase activation assay (ETKA); and high-performance liquid chromatography (HPLC) to assess TPP status, being a direct measurement of erythrocyte TPP. Specifically, the ETKA approach’s high sensitivity stems from erythrocytes being the first cells affected by low thiamine serum levels and their dependence on transketolase (TK) enzyme activity (Figure 4) [6].

In the cytosol, mitochondria, and peroxisome, thiamine pyrophosphate (TPP) is central to several metabolic processes. It serves as a cofactor for vital enzyme systems involved in carbohydrate, lipid, and branched-chain amino acid metabolism [4,19], and acts as a cofactor for transketolase within the pentose phosphate pathway (PPP) [8].

Specifically, transketolase enzyme (TK) plays a crucial role in regulating pentose metabolism, ensuring the production of the 5-carbon sugar ribose essential for nucleotide synthesis [3,18]: it indirectly affects NADPH synthesis, which is crucial for anabolic processes, and reduces natural antioxidants like glutathione (GSH) and ascorbic acid.

Maintaining the right transketolase activity is crucial for proper lipids, carbohydrates, and replication [4].

The crucial role of the pyruvate dehydrogenase complex (PDH) in energy production is highlighted by its catalysis of pyruvate to acetyl-CoA via oxidative decarboxylation. Therefore, this reaction links glycolysis to the tricarboxylic citric acid cycle (TCA), thus playing a crucial role in carbohydrate breakdown [3,8].

The mitochondrial respiratory chain’s complex I is directly linked to the Krebs cycle’s oxoglutarate dehydrogenase complex, the alpha-ketoglutarate dehydrogenase complex, or KGDHC. Three enzymes—dihydrolipoamide dehydrogenase (E3), dihydrolipoamide succinyltransferase (E2k), and α-ketoglutarate dehydrogenase (E1)—make up the multi-copy KGDHC complex. Specifically, the complex facilitates α-ketoglutarate’s conversion to succinyl-CoA via decarboxylation, generating nicotinamide adenine dinucleotide (NADH) and flavin adenine dinucleotide (FADH2), thus fueling adenosine triphosphate (ATP) production in the mitochondria [2,19].

The branched-chain α-keto dehydrogenase complex within the inner mitochondrial membrane catalyzes an irreversible oxidative decarboxylation of branched-chain alpha-keto acids to their corresponding short-chain acyl esters. The three branched-chain amino acids (BCAAs) L-isoleucine, L-valine, and L-leucine are categorized as essential amino acids with anabolic functions [2,3].

A multienzyme complex, branched-chain α-keto dehydrogenase, uses flavin adenine dinucleotide (FAD), coenzyme A, lipoamide, TPP, and NAD as cofactors and comprises a heterodimeric decarboxylase, a transacylase, and a homodimeric enzyme 3 components [2,3].

Peroxisomal 2-hydroxyacyl-CoA lyase (HACL1), which degrades fatty acids—including phytanic acid (3-methyl branched-chain fatty acids)—and shortens long-chain ones, uses TPP as a cofactor. The peroxisomal enzyme HACL1 uses TPP to process long-chain fatty acids through abbreviation and oxidation [2,8].

Thiamine’s antioxidant function, specifically its ROS scavenging, leads to a much greater reduction in hydroxyl radicals (HO•) compared to hydroperoxyl radicals (HOO•) [8]; in particular, thiamine supplementation is suggested to lower oxidative stress in heart failure (HF) by inhibiting the oxidation of oleic acid and lipid peroxidation. However, this might improve oxidative stress levels [6].

It also shows antioxidant effects in neutrophil cells, thereby supporting immunity. Decreasing oxidative-stress-induced NF-κB activation and modulating p53 activity via P43 inhibition safeguards macrophages, leading to anti-inflammatory effects [19].

Preliminary findings indicate a relationship between thiamine metabolism and cancer cell development [20].

Cancer cell response to thiamine supplementation is dose-dependent: low doses encourage growth, while high doses hinder it. Satisfying energy needs and increasing vital nutrient production for rapidly proliferating cancer cells is probably the primary cause of the effect. Cancer suppression results from pyruvate dehydrogenase kinase inactivation by high thiamine diphosphate. Pyruvate dehydrogenase complex inactivation, caused by phosphorylation from overactive pyruvate dehydrogenase kinases, is a process in cancer development. Conversely, thiamine diphosphate blocks kinase action, thus keeping pyruvate dehydrogenase complex activity at normal levels [4].

Thiamine exerts its effects by preventing pro-apoptotic Bcl-2 family protein expression, caspase-3 activation, and Poly (ADP-ribose) polymerase (PARP) cleavage—factors affecting cytochrome c release, mitochondrial membrane potential, AIF, and phosphorylation [3].

Furthermore, thiamine may inhibit advanced glycosylation end-products (AGEs) secretion, thus altering cellular function and structure, inducing apoptosis and protein changes, and causing tissue and organ damage. Specifically, reactive oxygen and nitrogen species produced by AGEs and their link to oxidative stress are the root causes of cardiovascular disease, diabetes, aging, and neurodegenerative diseases. Atherosclerosis development can be prevented through thiamine as it improves endothelial function and thus hinders atherosclerotic plaque formation [3].

## 2. Thiamine Accumulation

High thiamine intake or accumulation shows no adverse effects because the kidneys quickly remove excess thiamine through urine excretion; therefore, thiamine has no upper dosage limit [21].

Specifically, the transition between reabsorption and active secretion accelerates elimination. When this occurs, the glomerulus does not filter thiamine, leading to its excretion via renal tubular cells [17].

Generally, exceeding the recommended intake of thiamine orally is not usually associated with side effects. In contrast, though uncommon, parenteral thiamine treatment may lead to life-threatening complications, including anaphylaxis and cardiopulmonary arrest [10].

## 3. Thiamine Deficiency

Malnutrition, poor diet, diseases affecting the gut, surgery, alcoholism, diabetes, pregnancy, breastfeeding, and hyperthyroidism all increase the risk of thiamine deficiency, often due to higher metabolic needs or excretion (Figure 3) [13,22].

Furthermore, thiamine deficiency is frequently observed in those with alcoholism, cancer, or AIDS, much like post-bariatric-surgery patients; the reason is the short half-life and limited storage capacity of thiamine within the organism [19].

Many pathologies stemming from thiamine deficiency commonly appear in cardiovascular, neurological, and psychiatric systems. Thiamine-dependent biochemical pathway dysfunctions underlie the broad physiological impact on multiple systems [13].

Research indicates a high frequency of thiamine deficiency in the general population, with high-risk individuals exhibiting particularly high rates [4].

Healthy adults from middle- or high-socioeconomic groups with varied diets rarely experience thiamine deficiency [2].

Notably, thiamine deficiency is widespread in low- and middle-income nations, particularly those relying heavily on rice, such as Laos, Nepal, West Africa, Myanmar, Cambodia, and India [23].

Research indicates a thiamine diphosphate blood serum level of 57 nmol/l in Cambodian control mothers versus 126 nmol/l in American control mothers [4,24]; on the other hand, thiamine deficiency is a growing problem among children and adolescents in affluent nations [2].

According to this evidence, the 2003 epidemic of infant thiamine deficiency in Israel caused by thiamine-deficient infant formula is a clear sign of the world’s susceptibility to sudden thiamine depletion [2]. 

Thiamine deficiency is most common in infants and children and has a significant mortality rate [23]. The risk greatly multiplies if infants are solely breastfed and their mothers rely on refined grains for essential nutrients. During pregnancy, women might follow culturally unique intrapartum dietary restrictions, making them vulnerable to persistent hyperemesis, malabsorption, or other systemic illnesses. The impact of thiamine deficiency in breast milk is not apparent during early infancy because thiamine breaks down quickly [2].

According to a 1999 report by the World Health Organization, individuals with alcoholism often show thiamine deficiency in Europe, North America, and Australia. Some studies indicate that chronic alcoholics could have a thiamine deficiency of at least 25–31%, with figures reaching as high as 80% [19,25].

As our understanding of thiamine deficiency’s clinical signs advances, categorizing its manifestations becomes more challenging. The term ”thiamine deficiency disorders (TDDs)” was created to describe the symptoms resulting from thiamine deficiency at different stages of life [26].

Beriberi is classified as “wet” or “dry” based on cardiovascular symptoms, while Wernicke’s encephalopathy is the modern term for cognitive involvement [13,22].

An old legend from Sri Lanka tells of neurologically impaired patients being termed “beriberi” due to their restless movements, causing them to respond with “beriberi” in Sinhalese, meaning “I cannot”. Infants lacking thiamine were discovered to experience severe heart failure, commonly presented with wet lungs and swollen limbs regardless of their initial location or description [26].

There are two classifications for beriberi: “wet beriberi” for infants lacking thiamine and “dry beriberi” for older individuals with neurological issues. An infection alongside thiamine deficiency can worsen specific aspects of the illness [14].

Chronic amnesia, known as Korsakoff syndrome, develops from untreated Wernicke’s encephalopathy due to thiamine deficiency. Clinical signs of thiamine deficiency include feeling unwell, weight loss, urinary bladder incontinence, and rapid heartbeat. Anticholinergic autonomic dysfunction, confusion, and delirium are the hallmark symptoms of the classic triad in Wernicke’s encephalitis [27].

Wernicke’s encephalopathy, a manifestation of hypothiaminemia, affects 12.5–35% of individuals with alcoholism, compared to just 1.5% in the general population [10].

### 3.1. Causes of Thiamine Deficiency

As previously emphasized, thiamine deficiency often occurs in patients with various conditions [19], and its development could be associated with various factors, including furosemide usage, chronic kidney disease on hemodialysis, diabetes, gastrointestinal surgeries such as bariatric surgery, advanced HIV/AIDS, and alcoholism [10].

Moreover, individuals residing in regions with monotonous diets and eating low-thiamine starchy foods like refined rice or cassava are at risk of thiamine deficiency [23].

Deprivation risk is heightened by food insecurity from drought, famine, conflict, displacement, and severe acute malnutrition. Abnormal gut microbiota, like in those with severe acute malnutrition (SAM), could also lead to decreased thiamine absorption [23].

Consuming foods like tea leaves or betel nut, which contain thiamine antagonists, and African silkworm larvae or raw fish, which are thiamine oxidase inhibitors, may lead to thiamine deficiency [23].

Thiamine decomposition can occur in foods such as raw fish, coffee, tea, and shellfish due to enzymes, ultimately leading to thiamine depletion. Thiaminases play a role in breaking down thiamine molecules into inactive forms [28]. When thiamine levels decrease in food, Thiaminase I and Thiaminase II are both activated. Some bacteria and plants contain Thiaminase I, which has the ability to break down thiamine in food products; however, certain fish contain Thiaminase II, which has the ability to split the thiamine molecule into inactive pieces [17].

Understanding the role of thiaminases in causing thiamine deficiency in foods is crucial for maintaining adequate levels of this important nutrient in human diets [3].

Bariatric surgery increases the risk of thiamine deficiency due to impaired absorption, post-operative vomiting, and decreased vitamin intake, especially in patients who have had Roux-en-Y gastric bypass surgery. Specifically, thiamine deficiency can occur in these patients as a result of bypassing the small intestine where thiamine absorption is important [10].

The occurrence of TDDs is elevated in patients who undergo partial gastric surgery for cancer and peptic ulcer, along with bariatric surgery. Many research findings indicate that the reason for thiamine deficiency is unclear, possibly due to thiamine absorption in the duodenum, which may be affected by duodenal bacterial overgrowth or changes in acidity levels following gastrectomy [29].

Additionally, overweight individuals are at a higher risk of lacking thiamine. Typically, these patients have a diet that is deficient in vegetables and heavy in simple sugars and processed foods, which are deficient in thiamine [10].

Alcoholism is the main cause of thiamine deficiency, impacting various factors such as intake and enzyme activity in the small intestine [29].

Furthermore, alcohol consumption can reduce the caloric intake from non-alcohol sources, resulting in a decrease in thiamine consumption from the diet. Thiamine transport to the liver is additionally impaired by liver cirrhosis, a consequence of chronic alcohol abuse [30].

Thiamine deficiency can also be caused by higher urinary excretion, which can occur with increased urine volumes or prolonged use of loop diuretics. Dialysis can cause thiamine deficiency by removing it in severely ill patients on continuous renal replacement therapy [29].

Apart from furosemide, multiple drugs can boost the elimination of thiamine through urine, leading to a common deficiency in vitamin B1. Furthermore, several other drugs may influence thiamine dynamics. Nonetheless, the phosphorylation of thiamine into its active form TPP is blocked by the cytostatic drug 5-fluorouracil. Fedratinib—an anticancer agent inhibiting Janus kinase 2 (JAK2)—blocks the essential intestinal thiamine transporter THTR2 and plays a crucial role in the onset of Wernicke’s encephalopathy. Moreover, metformin results in a drug-vitamin interaction at THTR2 [9].

Thiamin deficiency has been linked to two rare genetic conditions, as indicated by certain evidence. The cause of Leigh’s syndrome is the destruction of thiamine diphosphate kinase, resulting in decreased thiamine triphosphate levels even when thiamine levels are adequate. A variation in thiamine-sensitive maple sugar urine disease affects children and can lead to developmental delays, nausea, learning disabilities, and lactic acidemia [29].

### 3.2. Diagnosis of Thiamine Deficiency

Thiamine deficiency is recognized when individuals have lower levels (ThDP) or activity (ETK) of thiamine compared to healthy individuals. A thiamine deficiency diagnosis is determined by laboratory tests, not by clinical signs or symptoms of the disease. Furthermore, the ThDP level and ETK activity coefficient are the main indicators of thiamine condition. The typical range of ThDP levels in whole blood for healthy individuals is 70 to 180 nmol/L. On the other hand, ETK activity indicates the role of thiamine in red blood cells. The ETK activity coefficient measures the change in ETK activity with exogenous ThDP supplementation. An elevated ratio above 1.25 could be a sign of thiamine deficiency but there is no unanimous decision [26].

A physician’s evaluation of a patient’s symptoms, possible thiamine deficiency in their diet, and response to treatment are all taken into account when diagnosing a TDD [7].

Excessive thiamine levels can complicate the adequacy hypothesis of certain test measures or reference intervals, influenced by experimental and clinical procedure variations. Furthermore, it is crucial to consider the investigation of subclinical alterations, whether genetic or metabolic, that may necessitate higher thiamine levels than what current testing parameters indicate [7].

### 3.3. Effects of Thiamine Deficiency on Metabolism

A lack of thiamine affects energy metabolism and the production of ATP [17].

Insufficient thiamine can hinder various metabolism-related enzymes and the Krebs cycle, causing reduced ATP synthesis, oxidative stress, and cell demise. Additionally, if thiamine deficiency persists, chronic energy deficiency can lead to severe malnutrition and weight loss [26].

Insufficient TPP causes pyruvate dehydrogenase to be inactive, blocking the conversion of pyruvate to acetyl CoA and the use of carbohydrates in the Krebs cycle. The build-up of pyruvate is redirected to the alternate anaerobic route of lactic acid formation, causing lactic acidosis and decreased mitochondrial ATP production [2].

Additionally, the increase in lactate levels results in a further decrease in mitochondrial function by obstructing the transport and breakdown of fatty acids and by reducing the function of carnitine palmitoyl transferases (CPT1/2) necessary for acylcarnitine uptake. The generation of reactive oxidative species could also be enhanced by lactate. Thiamine is crucial for producing nicotinamide adenine dinucleotide phosphate (NADPH) and nucleosides in the pentose phosphate pathway [29].

Thiamine deficiency can lead to a significant inhibition of the alpha-ketoglutarate dehydrogenase complex, causing a potential decrease in α-KGDH activity in just 4 days. In an experimental study using animal rodent models, thiamine deficiency led to a 52% decrease in enzyme activity in the sub-medial thalamic nucleus [2].

Reduced α-KGDH activity leads to elevated oxidative stress, excitotoxicity, lactate acidosis, neuronal death, inflammation, disrupted blood–brain barrier (BBB) permeability, glutamate buildup, and brain swelling [17].

Both thiamine deficiency and reduced TK levels can impair transketolase activity, possibly leading to a decrease within a week. Findings from mouse studies suggest that thiamine deficiency lowers transketolase activity, leading to impaired hippocampal neurogenesis and cognitive function [2].

Thiamine deficiency also affects enzyme complexes, including the branched-chain α-keto dehydrogenase complex and peroxisomal fatty acid metabolism, by altering enzyme metabolic activity [2].

### 3.4. Effects of Thiamine Deficiency on Cardiovascular System

The heart needs a significant amount of energy to perform its contracting and transporting functions [31]: ATP molecules play a key role in various processes such as cytoskeleton maintenance, calcium pumping, and muscle contraction in cardiomyocytes [32].

Nevertheless, the heart has a limited capacity to store energy, requiring significant power and depending largely on its mobile mitochondria that are involved in ATP production, lipid synthesis, and calcium balance.

Furthermore, cardiomyocytes need a continuous supply of energy, specifically, cardiac contraction relies on mitochondrial ATP [33].

The alteration in contractile function and the development of heart disease can be triggered by changes in cardiac metabolism, including substrate transformation and energy depletion [32].

The inhibition of key regulators due to thiamine deficiency results in impaired ATP production and compromised mitochondrial functions. TD could limit the production of ATP and acetyl-CoA [6].

Thus, a decreased release of ATP may also add to the energy depletion seen in HF patients, potentially impacting myocardial contractility and LVEF [6].

Thiamine deficiency disrupts regular heart function, and the breakdown of aerobic respiration processes hinders the heart’s normal operation. This is exacerbated by endovascular dysfunction from thiamine-dependent nitric oxide synthase (NOS), an enzyme found in the lungs of individuals with pulmonary hypertension. Specifically, thiamine is considered clinically important because its deficiency is linked to changes in myocardial antioxidant systems [34].

The progression of cardiovascular disease (CVD) is linked to endothelial dysfunction, a precursor to atherosclerosis triggered by persistent vascular inflammation, a significant risk factor for multiple heart conditions [35].

A previous study emphasized that clinical thiamine deficiency led to higher arteriovascular resistance. Specifically, thiamine positively impacts the growth of human intra-arterial smooth muscle cells by counteracting their proliferation due to glucose and insulin, playing a key role in atherosclerotic plaque advancement [11].

Moreover, thiamine mitigates the harmful effects of elevated glucose levels on endothelial cells through decreased protein glycation [36].

Thiamine has been found in multiple studies to have a preventative effect on inflammatory conditions, with thiamine levels showing an inverse relationship with chronic vascular inflammation and dyslipidemia [11].

Furthermore, thiamine deficiency is associated with heart hypertrophy, heart failure with preserved ejection fraction (HFpEF), and lactic acidosis (Cardiac and Shoshin beriberi) [37]. Cardiac arrhythmias are theoretically more likely with cardiomyocytic dysfunction from thiamine deficiency but this has only been observed experimentally in rats, and tachycardia is predominantly seen in infants [26].

Adults with cardiac involvement may experience edema in the lower extremities, liver, and spleen. The combination of ventricular dilation and pulmonary hypertension can lead to valve failure, particularly with regurgitation [26].

### 3.5. Effects of Thiamine Deficiency on HF

Thiamine is mainly transported to organs and tissues that have high metabolic needs and impacts high-metabolic systems such as the heart, muscles, nerves, and brain [15].

The cardiovascular system contains about half of the body’s thiamine concentration [38] and factors that lead to TD in HF include increased metabolic rate, older age, decreased appetite, frequent hospital stays, malnutrition, use of diuretics, HF severity, other health conditions, and insufficient thiamine intake [6].

Inadequate micronutrient levels might lower energy metabolism in heart muscle cells and cause harmful health consequences for CVD patients. Thiamine deficiency is commonly seen in people with heart failure and may impact clinical outcomes negatively [15].

Heart failure is a medical condition involving multiple systems, where the heart cannot pump enough blood to meet the body’s needs. Symptoms and signs of heart failure include trouble breathing when lying down, tiredness, breathlessness, swelling, fast heartbeat, and exercise intolerance [39].

Several metabolic abnormalities have been identified in the failing heart muscle, contributing to heart muscle dysfunction and the advancement of heart failure. Heart failure development is specifically associated with issues in aerobic oxidation, energy reserve depletion, and oxidative stress, impacting the heart’s pumping efficiency [6].

Lack of thiamine might amplify ATP production issues, resulting in decreased energy reserves and an elevated risk of heart problems [6].

The process of thiamine absorption and excretion in HF patients is intricate and diverges from the typical process. Individuals with heart failure may experience reduced thiamine intake due to feeling full earlier from cardiac cachexia and splanchnic congestion. HF patients should eliminate high-sodium thiamine sources from their diet. Moreover, patients with heart failure have increased thiamine needs because of prolonged diuretic usage, leading to potential kidney excretion [36].

Indeed, the potential reasons for thiamine deficiency in HF patients could include diuretic usage, metabolic alterations, and old age [11].

A lack of thiamine leads to decreased pyruvate dehydrogenase activity, causing pyruvate buildup and its conversion to lactate through anaerobic pathways. This process contributes to reduced peripheral resistance and increased venous return to the heart. The cause of congestive heart failure in thiamine deficiency is thought to be a mix of increased preload and myocardial dysfunction, according to some suggestions [5,40].

Pyruvate dehydrogenase relies on thiamine as an essential coenzyme. Thiamine deficiency causes wet beriberi and high-output heart failure by inhibiting this specific enzyme [41].

The lack of thiamine’s positive impact on metabolic processes is proposed as a possible cause of heart failure in beriberi, leading to a direct change in energy generation by the heart muscle. In this context, the primary signs of wet beriberi consist of increased heart rate (HR), heart enlargement, and excessive heart failure [5].

Furthermore, HF is typically recognized as a condition that primarily impacts older people. Thiamine deficiency is prevalent among the elderly, regardless of their heart condition. Individuals with heart failure tend to have especially elevated levels of thiamine deficiency [11].

Heart failure is a significant contributor to mortality and illness in Western nations. Approximately 6.5 million Americans suffer from heart failure, with close to 1 million new cases documented every year. Thiamine supplementation is suggested for severe thiamine deficiency causing high-output heart failure but its impact on heart failure patients overall is not yet clear. Due to heart failure being a key result of thiamine deficiency, various researchers have studied the possibility of using thiamine treatment in heart failure patients [5].

Physicians are advised by current guidelines to think about TD as a potential factor in HF based on the patient’s history and physical exam, even if thiamine levels are not usually tested in clinical practice [6]: it is crucial to study the significance of thiamine in preventing and treating both acute and chronic HF [38].

### 3.6. Nutritional Intervention to Improve Thiamine Status and Prevent Thiamine Deficiency

In regions where thiamine deficiencies are expected to be prevalent, strategies such as food fortification, supplementation, optimizing thiamine intake from diets, health education, and behavioral change are all significant for increasing thiamine intake [23].

The main strategy includes food fortification, a public health effort to boost thiamine levels in suitable food items. The effectiveness of biofortification via genetic modification in plants has been a subject of exploration [10].

Enhanced surveillance is crucial: specifically, nations with thiamine deficiency should require the evaluation of thiamine levels in pregnant women, young children, and infants, similar to the mandatory TSH test for infants, as well as in vulnerable populations [2].

The Beriberi Infant Project in Myanmar is a crucial intervention program where policy makers provide thiamine supplementation (10 mg/day) to pregnant and breastfeeding women. Nations with a high prevalence of thiamine deficiency are recommended to introduce this approach in addition to the existing compulsory iron and folic acid supplementation for expectant mothers [2].

In terms of nutrition, the recommendation is to address malnutrition and incorporate a variety of foods. To avoid thiamine deficiency, it is beneficial to eat a broad selection of foods, with an emphasis on legumes and vegetables [23].

Additionally, it is suggested to incorporate cooking practices that minimize the loss of thiamine in daily routines, for example, reduce pre-washing parboiled rice and cook with the perfect amount of water to avoid excess water use. Thus, to maintain food safety, it is recommended to avoid prolonged cooking periods, reheating dishes repeatedly, and storing uncooked foods for extended periods [23].

Deactivating substances that counteract thiamine could boost thiamine absorption. Properly cooking foods with thiaminase, like fish and meat, is crucial (heat destroys thiaminase). Improve absorption by refraining from tea, coffee, blueberries, and other polyphenol- and tannin-rich foods immediately after eating [2].

### 3.7. The Impact of Thiamine Deficiency: Molecular Mechanisms

Thiamine deficiency is thought to be connected to various cardiovascular diseases and related risk factors, such as type 1 and type 2 diabetes, obesity, chronic vascular inflammation, dyslipidemia, heart failure, myocardial infarction (MI), conduction defects, and depression [11]. Additionally, regarding the role of thiamine deficiency in the incidence and progression of bacterial endocarditis, there is only one scientific study dating back to 1985, which highlighted the decreased leucocyte mobility in patients with endocarditis and B1 deficiency (Table 1) [42].

Thiamine deficiency—also called “beriberi”—poses a major public health problem, especially in Asia, leading to significant mortality rates, especially among infants. Barennes et al. conducted a survey in 22 villages in North Laos and found that many children have a severe type of wet beriberi called Shoshin beriberi, leading to abnormally high lung pressures seen on 2D echocardiography [60]. Findings indicate a range of biomarkers—such as heme oxygenase (HO-1), superoxide dismutase (SOD), intercellular adhesion molecule 1 (ICAM-1), nitric oxide endothelial synthase (eNOS), nitric oxide inducible synthase (iNOS), ferritin, and malondialdehyde—increase in cases of thiamine deficiency [61]. A lack of thiamine hinders important controllers, affecting ATP synthesis and weakening mitochondrial activities [33]. This condition can slow the tricarboxylic acid cycle, further hindering ATP production and decreasing energy reserves, which is critical in the context of progressive heart failure (HF). As a result, a reduction in mitochondrial ATP levels can have a detrimental effect on the heart’s ability to contract and on the left ventricular ejection fraction (LVEF) [43], contributing to heart hypertrophy, HFpEF cardiac insufficiency, and lactic acidosis [44]. Furthermore, the pathophysiology of heart failure can significantly increase the risk of thiamine deficiency [40]. The development of high-output heart failure in beriberi is associated with an increased vasodilation of cardiac muscles and diminished peripheral vascular resistance, originating from impaired myocardial energy metabolism [62]. Animal studies have shown changes in heart size and function—as well as alterations in the heart cell structure and function—due to factors like reactive oxygen species (ROS) and decreased ATP levels. Nevertheless, there are opposing findings, emphasizing the need for further research. It is suggested that ion transportation methods might adapt in reaction to different heart-related issues such as thiamine deficiency [63]. In addition, reduced eNOS expression in major blood vessels results in significant endothelial dysfunction caused by thiamine deficiency. This reduction in contractility of cardiomyocytes leads to cardiac hypotrophy, bradycardia, and the onset of heart failure.

The activity of aortic valves did not decrease in response to thiamine deficiency, resulting in reduced acetylcholine-induced vascular relaxation as observed in the data. The findings demonstrated a heightened contractile response in aortas from rats with thiamine deficiency, which reverted to control response levels when the endothelium was removed [34] or NOS was inhibited with L-NAME (NG-nitro-L-arginine-methyl-ester). Thiamine deficiency involves metabolic disruptions that impact cardiovascular health, highlighting the need for thorough research and public health actions to tackle this crucial problem [11].

### 3.8. The Impact of Thiamine Deficiency and Furosemide Treatment on Cardiac Health

Chronic thiamine deficiency can cause significant changes in the myocardium, potentially resulting in arrhythmias. The clinical signs of this insufficiency include a larger heart, higher venous pressure, and acidosis, which may result in tachycardia in humans. Furosemide, a loop diuretic with a long history of use, is approved by the Food and Drug Administration (FDA) to manage volume overload and edema associated with congestive heart failure, liver failure, renal failure (RF), and nephrotic syndrome [64]. A follow-up study was conducted to assess thiamine levels in hospitalized patients with hypervolemic heart failure (HF) and/or RF who were being treated with furosemide. Despite the use of furosemide treatment, hypervolemic patients experienced a significant decrease in thiamine levels throughout their hospitalization. In addition, patients suffering from heart failure showed a notable decrease in thiamine levels. The thiamine levels of heart failure patients who had been prescribed oral furosemide before admission were further reduced [45]. Zhang et al. conducted additional research and discovered the SLC35F3 allele, responsible for encoding a thiamine transporter, and its link to disruptions in cardiac and autonomic functions [65]. The genetic study highlighted the role of the SLC35F3 gene in thiamine transport and its relationship with hypertension. By employing a genomic approach, subjects with extreme blood pressure values have been examined, underscoring the significance of SLC35F3 in multiple clinical, genetic, and biochemical contexts related to hypertension. The evidence supports the idea that SLC35F3 functions as a new thiamine transporter, indicated by decreased thiamine levels in living organisms and its capability to aid thiamine absorption in experimental conditions [65]. By indicating a role in thiamine transport, these results show that genetic variations in SLC35F3 can predict not just hypertension but also several cardiovascular and autonomic disorders. Despite these insights into the role of SLC35F3 in thiamine transport, uncertainties regarding its affinity and capacity remain. These data indicate that thiamine replacement could positively affect blood pressure, prompting the need for additional research to investigate this possibility [65]. Thus, understanding the interplay between thiamine transport, furosemide treatment, and cardiac health is crucial for improving outcomes in patients with heart failure.

### 3.9. The Role of Thiamine in Diabetes Mellitus: Implications of Deficiency for Metabolic Dysfunction

Thiamine deficiency is a prevalent issue in individuals with diabetes, potentially compounding the effects of hyperglycemia and contributing to metabolic dysfunction. Addressing thiamine status may offer a feasible approach to mitigate some complications associated with diabetes mellitus.

Carbohydrate metabolism critically depends on thiamine, whose deficiency exacerbates DM, a rising global health issue. DM is characterized by hyperglycemia, which can cause cellular damage through various mechanisms including endothelial dysfunction, increased oxidative stress, and the formation of AGEs [66,67].

Despite the unclear relationship between type 2 diabetes and thiamine deficiency, low thiamine levels in diabetic blood vessels could intensify metabolic problems with high blood sugar, severely impacting insulin creation and secretion. The distal nephrons excrete excess unbound protein thiamine, and loss of thiamine is closely related to renal clearance. An increase in renal clearance due to type 2 diabetes can therefore be linked to thiamine deficiency [49].

An additional study demonstrated that hyperglycemia and thiamine deficiency result in a reduction in the expression of THTR2 and transcription factor specificity protein-1 (Sp1), thereby impairing the transport of thiamine into glomerular cells. The cellular thiamine concentration heavily influences transketolase activity (a thiamine-dependent enzyme), emphasizing its crucial role in cell function. The microenvironment has a significant impact on glomerular endothelial cells, making them particularly vulnerable to glucose damage and thiamine deficiency [46].

Another study involving 120 adults with type 2 diabetes (T2D), including 46 individuals with microalbuminuria, found that thiamine deficiency was highly prevalent, occurring in 98% of patients with microalbuminuria and 100% of patients without microalbuminuria [47]. Additionally, lower blood thiamine levels were significantly associated with type 1 diabetes (T1D), inversely correlating with glucose levels compared to healthy individuals [11].

A comparison of metabolic variables and blood thiamine levels was conducted between patients with type 1 and type 2 diabetes and a healthy control group. Patients with type 1 and type 2 diabetes had significantly higher fasting blood sugar (FBS), random blood sugar (RBS), HbA1c, triglycerides, and total cholesterol than the control group. Furthermore, serum thiamine and high-density lipoproteins (HDLs) were considerably lower in both diabetic groups than in the control group. Across diabetic and control groups, FBS and HbA1c levels were strongly correlated [48].

## 4. The Benefits and Risks of Thiamine Supplementation

### 4.1. Thiamine Supplementation in Heart Failure

Regular thiamine consumption may lower the chances of developing hypertension, heart attacks, or angina; type 2 diabetes; depression; and high cholesterol. Adequate thiamine intake is correlated with lower HbA1c and fasting glucose levels compared to insufficient intake, as shown in the results [49].

Improving cardiac function, especially in elderly heart failure patients with low LVEF, may be achieved cost-effectively through thiamine supplementation [68]. Thiamine is considered to be a harmless drug and has not been linked to any major side effects even when taken at high doses of 300 to 900 mg daily [5]. Moreover, giving critically ill myocardial infarction (MI) patients in intensive care thiamine may reduce their risk of death [15].

The treatment of hospitalized acute heart failure patients with thiamine, in addition to standard care, is predicted to improve dyspnea. Acute heart failure was the primary diagnosis for the consecutive ER patients recruited over a two-year period. Participants received the study drug (100 mg thiamine or placebo) on the evening of days one and two. Thiamine levels increased significantly in the treatment group, while they remained unchanged in the control group. A significant difference in breath measurements over time between groups was only observed with oxygen, while sitting upright. Dyspnea and all secondary outcome measures showed no changes [41].

Dong and Wang investigated the link between thiamin and peripheral arterial disease (PAD) risk in over 6000 US adults [50]. Arteries outside the heart and brain can be affected by PAD, often a consequence of atherosclerosis [69].

A large percentage of people over 60 are affected by PAD, a common and serious condition that increases the likelihood of cardiovascular events and serious health problems [50]. Specifically, insufficient thiamine intake (less than 0.65 mg/day) correlated with a higher PAD risk. This implies a link between thiamine deficiency and a heightened risk of the condition. These findings could have an impact on dietary recommendations and prevention strategies for PAD, emphasizing the significance of adequate thiamine intake for cardiovascular health [50].

Higher lactate levels in hospitalized patients from clinical trials are associated with a greater chance of dying after cardiac arrest [70]. Interestingly, a clinical study investigated thiamine as a potential metabolic resuscitator for in-hospital cardiac arrest. The goal was to determine if thiamine could lower elevated lactate levels and enhance oxygen consumption in patients. Despite safety concerns causing the study’s termination after 36 participants, the preliminary data suggest that thiamine may substantially benefit cardiac arrest patients [51]. The application of thiamine in cardiac surgery was examined in other randomized trials [71].

Andersen et al. investigated post-operative lactate levels upon ICU arrival, also examining secondary outcomes including PDH activity, post-operative complications, ICU and hospital lengths of stay, and mortality. The findings suggest that giving thiamine could benefit patients having cardiac surgery by improving aerobic metabolism and lowering post-operative lactate [52]. Pre-surgical intravenous thiamine was typically used in these studies, with post-operative results assessed for enhanced carbohydrate metabolism [71]. Treatment with thiamine (400 mg/day), ascorbic acid (6 g), and hydrocortisone (200 mg/day) was found to significantly reduce vasopressor dependence and improve patient outcomes following vasoplegic syndrome after vascular surgery. By acting as a cofactor in lactate metabolism via lactate dehydrogenase, thiamine improves lactate clearance and reduces ascorbic acid conversion to oxalate, thereby preventing hyperoxaluria [53]. In addition, clinical evidence suggests the potential role of thiamine supplementation in patients with endocarditis. The efficacy of intravenous thiamine on oxygen consumption (Vo2) was assessed in a prospective, open-label pilot study of critically ill adults. Patients needing mechanical ventilation and having diagnoses such as endocarditis, pancreatitis, pleural effusion, or cardiac arrest were screened for the study [59]. Oxygen consumption (Vo2) and the cardiac index were measured continuously for 9 h and, in particular, three hours after the baseline data collection, 200 mg of thiamine was injected intravenously. After thiamine administration, an increase in Vo2 (16.3 mL/min, SE 8.5; *p* = 0.052) occurred, and for patients presenting with an average cardiac index greater than the cohort’s mean value of 3 L/min/m^2^, Vo2 increased by 70.9 mL/min (*p* < 0.0001) after thiamine. Thiamine had no effect in patients with a reduced cardiac index (<2.4 L/min/m^2^) and no association between the initial thiamine level and changes in Vo2 after thiamine administration was observed. Nevertheless, due to the administration of a single dose of thiamine, an increase in Vo2 in critically ill patients and a considerable increase in Vo2 in patients with a preserved or elevated cardiac index was observed [59].

### 4.2. The Potential Benefits of Thiamine Supplementation in Diabetes Management

Observations on thiamine supplementation in diabetes have highlighted its potential for mitigating disease complications, attracting interest. Recent studies show that thiamine can lessen high glucose’s negative impact on endothelial cells [71]. Six months of 100 mg thiamine supplementation could help T2DM patients by addressing thiamine deficiency and improving lipid and creatinine levels. Significant improvements in T2DM patients’ metabolic profiles, including serum thiamine, lipid, and creatinine levels, were observed following oral thiamine supplementation in a brief interventional study [54]. Although kidney function markers were not fully assessed, a significant drop in serum creatinine—a key biomarker—was observed [54].

Due to its effectiveness against macrovascular disease and low cost, metformin is the most common first treatment for type 2 diabetes [72]. However, metformin-associated lactic acidosis, though rare, is a potentially life-threatening complication in diabetes treatment [73]. Patients with chronic kidney disease, congestive heart failure, or a history of alcohol consumption, severe dehydration, shock, hypoxia, sepsis, or advanced age face a heightened risk of biguanide-associated lactic acidosis [72]. Severe lactic acidosis in patients taking biguanides necessitates immediate high-dose thiamine treatment to mitigate the risk of thiamine deficiency, according to studies. Starting treatment right away is essential when a thiamine deficiency is suspected since supplementation leads to a rapid improvement in the patient’s health [74].

In a small clinical trial involving patients with gestational diabetes, thiamine supplementation over a period of 6 weeks was found to reduce levels of specific markers of inflammation and oxidative stress, including serum C-reactive protein, tumor necrosis factor-alpha (TNF-α) gene expression, and plasma malondialdehyde (MDA) levels [55].

A further investigation explored the effects of thiamine supplementation on inflammatory and oxidative stress biomarkers in those with gestational diabetes. Thiamin supplementation significantly decreased serum high-sensitivity C-reactive protein (hs-CRP) and plasma MDA levels when compared with the placebo. Supplementation with thiamin lowered TNF-α gene expression, specifically in the peripheral blood mononuclear cells of GDM patients; however, other inflammatory and oxidative stress biomarkers remained unaffected [55].

Furthermore, a 26-year-old woman with childhood-onset diabetes mellitus presented with a clinical case of thiamine-responsive megaloblastic anemia (TRMA) [56]. Genetic testing, prompted by the patient’s visual disturbances and anemia, revealed an SLC19A2 gene mutation, thus confirming a diagnosis of TRMA. After beginning thiamine treatment, the patient showed significant improvement in hemoglobin levels and glycemic control but the retinal damage caused by rod-cone dystrophy was found to be permanent [56]. The study highlights the significance of early diagnosis coupled with thiamine treatment in enhancing the life quality of individuals diagnosed with TRMA; however, it acknowledges thiamine’s ineffectiveness in averting hearing loss. The need for high clinical suspicion to diagnose TRMA and the importance of timely intervention are highlighted [56].

### 4.3. The Beneficial Effects of Thiamine Supplementation in Atherosclerosis Development and Progression

Diabetes mellitus increases the risk and speed of developing atherosclerosis and cardiovascular disease. The morbidity and mortality associated with diabetes are significantly impacted by diabetic microvascular and macrovascular dysfunction [75]. Early atherosclerosis is characterized by the impairment of endothelium-dependent vasodilation (EDV) [76].

This finding likely has significant clinical implications, especially in populations where cardiovascular disease is a leading cause of death and disability. This is especially relevant for patients with impaired glucose tolerance (IGT) and non-insulin-dependent diabetes mellitus (NIDDM), who are prone to accelerated atherosclerosis [57]. Improved endothelial function, and a resulting slower progression of atherosclerosis, may occur with routine thiamine administration.

Attenuated brachial artery vasoactivity (BAVA) signals arterial disease due to endothelial dysfunction, a process thought to begin early in atherosclerosis. Due to thiamine’s vasodilatory effects and importance in glucose metabolism, a study will assess its effect on BAVA during acute hyperglycemia [57]. Thiamine was suggested as a preventative measure against impaired glucose tolerance across various groups, including healthy people, those with reduced glucose tolerance, and early-stage NIDDM patients experiencing hyperglycemia [57]. Other studies show that vitamin B1 supplements significantly reduce vascular inflammation; high thiamine levels correlate negatively with LDLc and triglycerides. Therefore, it is possible to consider that the progression of atherosclerosis can be slowed down through chronic vitamin B1 administration [54].

**Figure 5:**
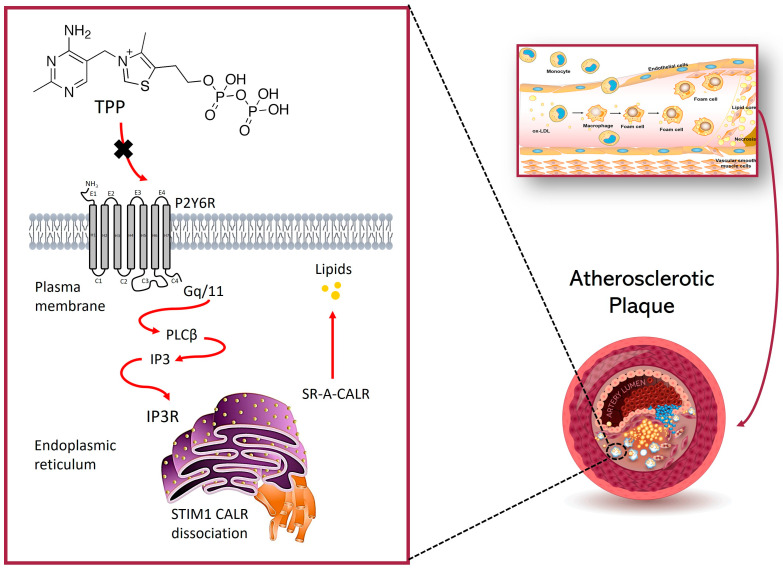
Foaming cells and atherosclerosis can be regulated by the P2Y6 receptor in macrophage cells, which can contribute to the signaling of CALR. P2Y6 inhibition by thiamine pyrophosphate prevents phospholipase Cβ from signaling and STIM1 entry. By decreasing the binding between the A-receptor of the eater and calreticuline (CALR), foamed cells are reduced. Thiamine pyrophosphate (TPP); phospholipase Cβ (PLCβ), IP3, inositol 1,4,5-trisphosphate receptor (IP3R); scavenger receptor A (SR-A); calreticuline (CALR); P2Y6 receptor (P2Y6R); Stromal Interaction Molecule 1 (STIM1); oxidized low-density lipoprotein (ox-LDL).

Furthermore, the pyrimidinergic receptor (P2Y6) in macrophages may regulate calreticuline (CALR) signaling, impacting analyzer A protein levels and thus contributing to foam cell formation and atherosclerosis. The findings indicated that P2Y6 deficiency suppresses phospholipase Cβ and Stromal Interaction Molecule 1 (STIM1)-mediated calcium influx [77]. This leads to a decrease in the binding between the A-receptor of the eater and CALR, a change that contributes to the reduction in foaming cells. This indicates P2Y6 may be a viable therapeutic target for atherosclerotic diseases, treatable with antagonists like thiamine pyrophosphate. Thiamine pyrophosphate exhibits remarkable activity and binding affinity as a potent inhibitor of the P2Y6 receptor (Figure 5). This indicates its potential as an atherosclerosis drug [77].

### 4.4. Supplementation of Lipophilic Derivatives of Thiamine and Their Therapeutic Potential

Allithiamine, derived from garlic (genus Allium), represents the first identified lipophilic thiamine derivative. The synthesis of several lipophilic analogs yielded fursultiamine, sulbutiamine, and benfotiamine [78]. In several conditions, especially those linked to high blood sugar and oxidative stress, benfotiamine has demonstrated considerable therapeutic potential. A key difference between these two is their effectiveness in dealing with issues caused by high blood sugar [22]. Early findings suggest that benfotiamine could be effective in preventing endothelial dysfunction following meals in patients with type 2 diabetes. Thirty-one patients with type 2 diabetes participated in a double-blind, randomized, placebo-controlled crossover trial, receiving either 900 mg/day of benfotiamine or a placebo for six weeks. The basal flow-mediated dilation was impaired in participants. After placebo, high baseline flow-mediated dilation patients experienced a significant postprandial decrease; benfotiamine pre-treatment, however, countered this (Table 2) [58].

#### 4.4.1. Thiamine Pyrophosphate (TPP) Supplementation

Regarding thiamine pyrophosphate (TPP), an endothelial cell protectant [84], researchers studied NO synthesis in endothelial cells exposed to TPP and high glucose. Cell selection from newborn umbilical cord veins is careful, accounting for family history of diabetes or hypertension, known to influence the endothelial response. According to the results, 0.625 mg/mL of TPP in the presence of 5 mmol/L of glucose does not affect the viability or proliferation of endothelial cells. However, the viability and proliferation of endothelial cells increased when incubated with TPP and high glucose. To conclude, the observed effects of TPP on glucose and NO suggest its protective mechanism in the endothelium of patients with diabetes [79].

Additionally, research has demonstrated variability in TPP levels. For example, cellular pyrimidine depletion reduces synthesis—a reaction performed by TPP kinase 1 (TPK1) —which reportedly phosphorylates thiamine using ATP. Furthermore, it has been revealed that TPK1 preferentially utilizes uridine 5′-triphosphate (UTP), leading to cellular TPP synthesis, activation of PDH and the TCA cycle, lipogenesis, and adipocyte differentiation. Thus, UTP is necessary for the utilization of vitamin B1 to maintain pyruvate oxidation and lipogenesis [85].

#### 4.4.2. Allithiamine Supplementation

A study modeled hyperglycemia’s effect on endothelial cells using human umbilical vein endothelial cells (HUVECs) to assess the impact of allithiamine, a less-polar thiamine derivative from garlic [80]. The main results show that allithiamine, purified and verified by techniques such as matrix-assisted laser desorption/ionization mass spectrometry (MALDI-MS) and high-performance liquid chromatography–tandem mass spectrometry (HPLC-MS/MS), has significant antioxidant effects, reducing the production of ROS and suppressing the increase in AGEs induced by hyperglycemia. The activation of NF-κB, a crucial inflammation mediator, was negatively impacted by allithiamine, suggesting its possible use in managing diabetes-related vascular complications [80].

### 4.5. The Role of Thiamine in Cancer Therapy and Cardiotoxicity Prevention

**Figure 6:**
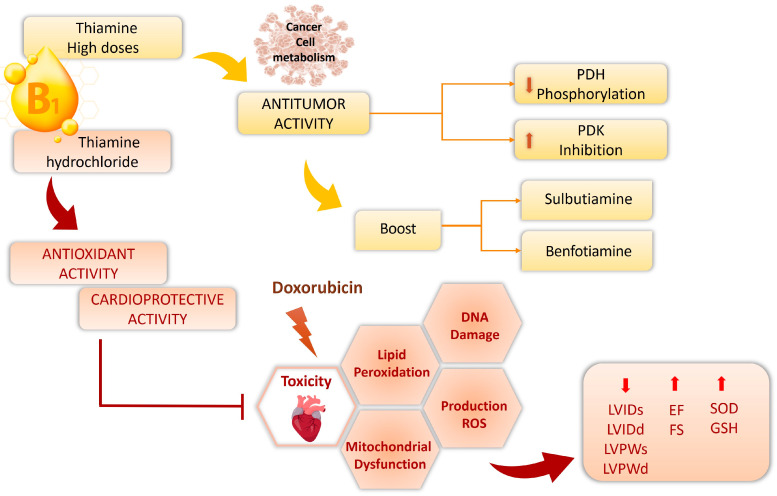
Thiamine supplementation may influence cancer cell metabolism by acting as a cofactor for their elevated metabolic rate. Thiamine exhibits antitumor effects by potentially reducing PDH phosphorylation and enhancing its enzymatic activity through PDK inhibition. The anticancer properties of thiamine are enhanced by using commercially available lipophilic analogs such as sulbutiamine and benfotiamine. Moreover, thiamine hydrochloride has strong cardioprotective and antioxidant properties and significantly reduces the damage caused by DOX treatment, including lipid peroxidation, DNA damage, mitochondrial dysfunction, and ROS production, with a subsequent reduction in echocardiographic parameters (LVIDs, LVIDd, LVPWs, and LVPWd) along with increased levels of antioxidants like SOD and GSH and improved EF and FS. Pyruvate dehydrogenase complex (PDH); pyruvate dehydrogenase kinase (PDK); left ventricle internal dimension at end-systole (LVIDs); left ventricle internal dimension at end-diastole (LVIDd); left ventricular posterior wall thickness at end-systole (LVPWs); left ventricle internal dimension at end-diastole (LVPWd); superoxide dismutase (SOD); glutathione (GSH); ejection fraction (EF); fractional shortening (FS); reactive oxygen species (ROS); doxorubicin (DOX).

Supplemental vitamins may affect cancer cell metabolism by providing extra nutrients that act as cofactors for their increased metabolic rate (Figure 6) [86].

High-dose thiamine shows antitumor effects by potentially reducing PDH phosphorylation and boosting its enzymatic activity via pyruvate dehydrogenase kinase (PDK) inhibition. Despite its high bioavailability potentially limiting its clinical use, thiamine is still a promising nutraceutical option for cancer treatment [81]. Thiamine’s in vitro anticancer action is amplified by using commercially available lipophilic analogs including sulbutiamine and benfotiamine. Sulbutiamine and benfotiamine failed to lower the thiamine’s half-maximal inhibitory concentration (IC50) value from millimolar to micromolar concentrations. HPLC analysis showed that sulbutiamine and benfotiamine significantly impacted intracellular thiamine and TPP levels in vitro, decreasing PDH phosphorylation [81]. The drug doxorubicin (DOX) shows effectiveness against a wide range of malignancies. The serious side effect of cardiotoxicity from anthracycline is clinically significant in cancer therapy [87]. The protective effects of thiamine (25 mg/kg i.p.) against DOX-induced cardiotoxicity were investigated in rats. Cardiac structure preservation following DOX—as indicated by reduced left ventricle internal dimension (LVIDs) at end-diastole (LVIDd), left ventricular posterior wall thickness at end-systole (LVPWs), and left ventricle internal dimension at end-diastole (LVPWd)—was achieved with thiamine pre-treatment. Additionally, the DOX+THIA group presented an improvement in EF and FS relative to the DOX group of rats. Compared to DOX-treated rats, 7 days of thiamine treatment significantly boosted SOD and GSH antioxidant levels in heart tissue. Thiamine’s ability to restore cardiac tissue’s ROS/antioxidant balance after a single DOX injection confirms its potent antioxidant and cardioprotective effects [82].

A further study from Radonjic and colleagues examined thiamine hydrochloride’s cardioprotective effects against DOX-induced cardiotoxicity, contrasting outcomes with and without prior thiamine administration. Parameters such as the maximum rate of left ventricular development (dp/dt max), minimum rate of left ventricular development (dp/dt min), systolic left ventricular development (SLVP), diastolic left ventricular development (DLVP), HR, and coronary flow (CF), pro-oxidative and antioxidative markers, cardiac activity, and a histopathological evaluation were measured [83]. Thiamine pre-treatment reduced the significant changes in cardiac contractility caused by DOX treatment. The findings suggest that giving thiamine hydrochloride before DOX may decrease oxidative stress, increase myocardial contractility, and enhance antioxidant defenses [83].

## 5. Conclusions

To sum up, this comprehensive review aimed to highlight the importance of vitamin B1 in an overall healthy basal diet to promote the prevention of cardiovascular dysfunctions. Preclinical and clinical thiamine supplementation studies show significant B1 vitamin impacts in managing cardiovascular disease.

Thiamine, notably, is found in the human body in different forms; these include thiamine monophosphate, thiamine diphosphate, and thiamine triphosphate [3]. Several factors—including poor nutrition, digestive problems, alcohol abuse, cancer, diabetes, pregnancy, breastfeeding, and an overactive thyroid—can rapidly cause thiamine deficiency [13,22]. Thiamine concentrations in humans are affected by genetic defects that alter thiamine transporters. Genetic mutations causing SLC19A2 dysfunction can specifically result in outcomes such as megaloblastic anemia (TRMA), sensorineural hearing loss, hyperglycemia, and diabetes mellitus [16]. Hyperglycemia and metabolic issues in diabetics are potentially worsened by a common thiamine deficiency. Thiamine deficiency can impair energy metabolism, ATP production, and various enzymes related to metabolism, leading to reduced ATP synthesis, oxidative stress, and cell death. Furthermore, unaddressed thiamine deficiency results in severe malnutrition and weight loss from long-term energy deficits [26]. Pyruvate dehydrogenase inactivity, due to insufficient TPP, blocks pyruvate-to-acetyl CoA conversion and carbohydrate use within the Krebs cycle. Pyruvate accumulation diverts to lactic acid production anaerobically, leading to lactic acidosis and reduced mitochondrial ATP synthesis [2].

Persistent vascular inflammation is a major risk factor for many heart problems, causing endothelial dysfunction which leads to atherosclerosis and CVD progression [35].

The negative effects of high glucose on endothelial cells are lessened by thiamine’s mitigation of protein glycation [36]. In fact, carbohydrate metabolism relies heavily on thiamine and a lack of it intensifies diabetes and all consequences of hyperglycemia, such as endothelial dysfunction, oxidative stress, and AGE formation, leading to cellular damage [66,67].

Regular thiamine intake may indeed reduce the risk of hypertension, heart attacks, angina, type 2 diabetes, high cholesterol, and depression. The evidence highlighted a correlation between adequate thiamine intake and lower HbA1c and fasting glucose levels, in contrast to insufficient intake [49].

Thiamine supplementation might provide a cost-effective way to improve cardiac function, particularly for older heart failure patients with a reduced LVEF [68]. The potential of thiamine to reduce diabetes complications is highlighted by observations, leading to increased interest. Recent research indicates thiamine may mitigate high glucose’s adverse effects on endothelial cells.

Furthermore, the accelerated atherosclerosis (impaired endothelium-dependent vasodilation and cardiovascular disease) associated with diabetes mellitus suggests that improved glycemic control could substantially impact microvascular and macrovascular dysfunction [75,76]. Routine thiamine may lead to better endothelial function and slower atherosclerosis progression. This finding has the potential to greatly influence healthcare, especially where cardiovascular issues are common. Moreover, supplementing with thiamine may affect cancer cell metabolism, given its role as a cofactor in their rapid metabolism [82]. This could also lessen doxorubicin-related cardiotoxicity (including lipid peroxidation, DNA damage, mitochondrial dysfunction, and ROS generation), improving echocardiogram parameters [83].

In conclusion, despite promising preclinical thiamine data, clinical trials show conflicting results due to small sample sizes and insufficient follow-up. More research is necessary to explore thiamine’s potential advantages, address current study limitations, and assess its use as an adjunct therapy with standard treatments for various high-cardiovascular-risk conditions. Indeed, observational data from a recent large cohort clinical study indicated a possible association between greater vitamin B1 intake and a decreased risk of hypertension, heart failure, and cardiovascular mortality [88]. In particular, more pronounced protective effects from thiamine supplementation could be achieved in elderly men, overweight people, smokers, drinkers, and in patients suffering from hyperglycemia and dyslipidemia.

## Figures and Tables

**Table 1:** Thiamine and cardiometabolic disease management in clinical trials.

	The Impact of Thiamine Deficiency: Molecular Mechanisms		
Author, Year	Clinical Trials	Properties	Ref.
Keith M. et al., 2019 Yamada Y. et al., 2021	Depletion of mitochondrial ATP resulting from thiamine deficiency	Negatively impacts myocardial contractility and LVEF; Contributes to heart hypertrophy, HFpEF cardiac insufficiency, and lactic acidosis.	[43,44]
Gioda C.R. et al., 2014	Thiamine deficiency induces significant endothelial dysfunction and a reduction in the contractility of cardiomyocytes	Decreases expression levels of eNOS in highly conductive blood vessels; Development of cardiac hypotrophy, bradycardia, and the onset of heart failure.	[34]
Gioda C.R. et al., 2014	NO measurements in a thiamine deficiency study	Decrease in the activity of aortic valves, associated with a reduction in acetylcholine-induced vascular relaxation. The results demonstrated a heightened contractile response in aortas from rats with thiamine deficiency, which reverted to control response levels when the endothelium was removed or NOS was inhibited with L-NAME.	[34]
	**The Impact of Thiamine Deficiency and Furosemide Treatment on Cardiac Health**		
**Author, Year**	**Clinical Trials**	**Properties**	**Ref.**
Bicer I. et al., 2023	The investigation aimed to delineate thiamine status in hospitalized patients with hypervolemic HF and/or RF receiving furosemide treatment	Thiamine levels in hypervolemic patients showed a significant decline during hospitalization, despite continued furosemide treatment (*p* = 0.029); A significant decrease in thiamine levels was observed specifically in patients with HF (*p* = 0.026).	[45]
	**The Role of Thiamine in Diabetes Mellitus: Implications of Deficiency for Metabolic Dysfunction**		
	**Clinical Trials**	**Properties**	**Ref.**
Mazzeo A. et al., 2021	Clinical study about the association of hyperglycemia and thiamine deficiency	Reduction in the expression of THTR2 and Sp1, thereby impairing the transport of thiamine into glomerular cells.	[46]
Nix W.A. et al., 2015	Study involving 120 adults with T2D, including 46 individuals with microalbuminuria	Thiamine deficiency was highly prevalent, occurring in 98% of patients with microalbuminuria and in 100% of those without it.	[47]
Eshak E.S. et al., 2018 Al-Daghri N.M. et al., 2015	Investigation into individuals with T1D	Presented lower blood levels of thiamine compared to healthy controls, with thiamine levels inversely correlating with glucose levels.	[11,48]
Al-Daghri N.M. et al., 2015	Blood thiamine levels in patients with type 1 and type 2 diabetes were evaluated, comparing them to a healthy control group	Increased levels of FBS, RBS, HbA1c, triglycerides, and total cholesterol compared to controls; Notably lower serum thiamine and HDL levels than the controls.	[48]
	**Supplementation in Heart Failure**		
**Author, Year**	**Clinical Trials**	**Properties**	**Ref.**
Duc H.N. et al., 2021	According to research, daily intake of thiamine	Reduce risks of hypertension (OR 0.95; 95% CI 0.90, 0.99), myocardial infarction, angina (OR 0.84; 95% CI 0.74, 0.95), type 2 diabetes (OR 0.86; 95% CI 0.81, 0.93), depression (OR 0.90; 95% CI 0.83, 0.97), and dyslipidemia (OR 0.90; 95% C0.86, 0.95); HbA1c and fasting glucose levels were lower in participants with adequate thiamine intake than those with insufficient intake.	[49]
Smithline H.A. et al., 2019	The study included hospitalized patients with acute heart failure, supplementing thiamine with the standard of care to improve dyspnea	Thiamine levels increased significantly in the treatment group, while they remained unchanged in the control group; A significant difference between groups over time of breath measured in the upright sitting position with oxygen; No changes were observed for other measures of dyspnea and for all secondary measures.	[41]
Dong Z. et al., 2024	Dong and Wang’s study included over 6000 participants and aimed to examine the relationship between thiamin intake and PAD risk among US adults	Thiamine intake below 0.65 mg/day was found to be associated with an increased risk of developing PAD; Insufficient levels of thiamine may contribute to an increased vulnerability to this condition.	[50]
Berg K.M. et al., 2024	The clinical study investigated thiamine as a potential metabolic resuscitator for in-hospital cardiac arrest to determine if thiamine could lower elevated lactate levels and enhance oxygen consumption in patients	Existing research indicates that thiamine may significantly improve outcomes for cardiac arrest survivors, although the study was halted after enrolling 36 patients due to safety concerns.	[51]
Andersen L.W. et al., 2016	Andersen et al.’s research analyzed post-operative lactate levels at the time of arrival in intensive care	Thiamine administration could improve aerobic metabolism and decrease post-operative lactate levels; The study looked at secondary endpoints like PDH activity, post-operative complications, duration of intensive care and hospital stay, and mortality.	[52]
Datt V. et al., 2021	The study investigates vasoplegic syndrome following vascular surgery, administering thiamine (400 mg/day) in combination with ascorbic acid (6 g) and hydrocortisone (200 mg/day)	Significant reduction in the need for vasopressors and offers benefits in terms of both mortality and morbidity; Thiamine decreased the conversion of ascorbic acid to oxalate, preventing hyperoxaluria, and improved the clearance of lactate by serving as a cofactor in the metabolism of lactate-by-lactate dehydrogenase.	[53]
	**Thiamine Supplementation in Diabetes Management**		
**Author, Year**	**Clinical Trials**	**Properties**	**Ref.**
Al-Attas O. et al., 2014	Interventional follow-up study based on supplementing with 100 mg of thiamine for six months for patients with T2DM	Increased serum thiamine levels and its derivatives, as well as enhancements in lipid and creatinine levels; A significant decrease in serum creatinine levels was noted over time, which is a reliable indicator of kidney function.	[54]
Amirani E. et al., 2022	A small clinical trial that involved patients with gestational diabetes who received thiamine supplementation for a period of 6 weeks	Decreased levels of specific markers of inflammation and oxidative stress, including serum C-reactive protein, TNF-α gene expression, and plasma MDA levels.	[55]
Amirani E. et al., 2022	A clinical investigation was carried out to evaluate the effects of thiamin supplementation on biomarkers of inflammation and oxidative stress in patients with gestational diabetes mellitus (GDM)	Significantly decreased serum hs-CRP (β—0.98 mg/L; 95% CI, −1.54, −0.42; *p* = 0.001) and plasma MDA levels (β—0.86 µmol/L; 95% CI, −1.15, −0.57; *p* < 0.001) when compared with the placebo; downregulation of gene expression of tumor necrosis factor-alpha (TNF-α) (*p* = 0.002) in peripheral blood mononuclear cells of patients with GDM; Not affecting other biomarkers of inflammation and oxidative stress.	[55]
Veetil V.M. et al., 2023	A clinical case of thiamine-responsive megaloblastic anemia (TRMA) in a 26-year-old woman who was diagnosed with diabetes mellitus in childhood	The patient showed significant improvement in hemoglobin levels and glycemic control; The study emphasizes the importance of thiamine treatment to improve the quality of life for patients with TRMA, while noting that thiamine cannot prevent hearing loss.	[56]
	**Thiamine Supplementation in Atherosclerosis**		
**Author, Year**	**Clinical Trials**	**Properties**	**Ref.**
Arora S. et al., 2006	The study has been proposed to evaluate the impact of thiamine on BAVA when there is acute hyperglycemia	Thiamine would protect against impaired glucose tolerance in healthy individuals, those with reduced glucose tolerance, and those with early NIDDM in hyperglycemic conditions.	[57]
Al-Attas O. et al., 2014	Evaluation of vitamin B1 supplementation and related hypolipidemic effect	Significant effect on reducing vascular inflammation, and there are negative correlations between high levels of thiamine and LDLc, as well as triglycerides; The progression of atherosclerosis can be slowed down through chronic vitamin B1 administration.	[54]
	**Supplementation of Lipophilic Derivatives of Thiamine and Their Therapeutic Potential**		
**Author, Year**	**Clinical Trials**	**Properties**	**Ref.**
Stirban A. et al., 2013	In a placebo-controlled randomized double-blind crossover trial, 31 individuals with type 2 diabetes were given either 900 mg/day benfotiamine or a placebo for a 6-week period	The basal flow-mediated dilation was impaired (2.63 ± 2.49%) in participants; After placebo treatment, postprandial flow-mediated dilation decreased significantly in patients with the highest flow-mediated dilation but benfotiamine pre-treatment mitigated this effect.	[58]
	**The Role of Thiamine in Preventing Cardiac Inflammation**		
**Author, Year**	**Clinical Trials**	**Properties**	**Ref.**
Berg K.M. et al., 2014	In a pilot, open-label, prospective study, patients with different diagnoses—including endocarditis, pancreatitis, pleural effusion, and cardiac arrest—were injected intravenously with 200 mg of thiamine	No effects were observed in patients with reduced cardiac index (< 2.4 L/min/m^2^). No association was observed between initial thiamine level and change in Vo2 after thiamine administration. An increase in Vo2 in critically ill patients and a considerable increase in Vo2 in patients with preserved or elevated cardiac index were observed.	[59]

ATP: adenosine triphosphate; CI: confidence interval; LVEF: left ventricular ejection fraction; HFpEF: heart failure with preserved ejection fraction; eNOS: endothelial nitric oxide synthase; NO: nitric oxide; NOS: nitric oxide synthase; L-NAME: L-NG-Nitroarginine Methyl Ester; HF: heart failure; RF: renal failure; THTR2: thiamine transporter 2; Sp1: transcription factor specificity protein-1; T2D: type 2 diabetes; T1D: type 1 diabetes; FBS: fasting blood sugar; RBS: random blood sugar; HbA1c: glycated hemoglobin; HDLs: high-density lipoproteins; OR: odds ratio;; PAD: peripheral arterial disease; US: United States; PDH: pyruvate dehydrogenase; T2DM: type 2 diabetes mellitus; TNF-α: tumor necrosis factor-alpha; MDA: malondialdehyde; GDM: gestational diabetes mellitus; hs-CRP: high-sensitivity C-reactive protein; TRMA: thiamine-responsive megaloblastic anemia; BAVA: brachial artery vasoactivity; NIDDM: non-insulin-dependent diabetes mellitus; LDL: low-density lipoprotein; Vo2: oxygen consumption.

**Table 2:** In vitro and in vivo evidence of thiamine-induced beneficial effects.

Supplementation of Lipophilic Derivatives of Thiamine and Their Therapeutic Potential		
In Vitro and In Vivo Studies	Properties	Ref.
Some researchers have evaluated the synthesis of NO in endothelial cells incubated with TPP and high concentrations of glucose, considering their family history since a history of diabetes or hypertension has been linked to endothelium response.	According to the results, 0.625 mg/mL of TPP in the presence of 5 mmol/L of glucose does not affect the viability or proliferation of endothelial cells; However, incubating endothelial cells with TPP and a high glucose concentration increased their viability and proliferation, the presence of TPP regulates the consumption of glucose and the synthesis of NO, which would explain its protective effect in the endothelium of diabetic patients.	[79]
The study examines the effects of garlic-derived allithiamine, a derivative of thiamine (B1) with less polarity, on hyperglycemic-induced endothelial dysfunction using HUVECs as a model for hyperglycemia.	Allithiamine has significant antioxidant effects, reducing the production of ROS and suppressing the increase in AGEs induced by hyperglycemia; It also negatively affected the activation of NF-κ B.	[80]
**The Role of Thiamine in Cancer Therapy and Cardiotoxicity Prevention**		
**In Vitro and In Vivo Studies**	**Properties**	**Ref.**
The study evaluated the antitumor effect of thiamine in vitro, taking advantage of commercially available lipophilic thiamine analogs, such as sulbutiamine and benfotiamine, which enhance the antitumor effect.	Neither sulbutiamine nor benfotiamine decreased thiamine’s millimolar IC50 value to micromolar equivalents; According to HPLC analysis, sulbutiamine and benfotiamine had a significant effect on the intracellular concentrations of thiamine and TPP in vitro, leading to a reduction in PDH’s phosphorylation levels.	[81]
The study investigated the protective role of thiamine (25 mg/kg i.p.) against DOX-induced cardiotoxicity in rats.	Thiamine pre-treatment preserved cardiac structure after DOX application in terms of lowering LV dimensions LVIDs, LVIDd, LVPWs, and LVPWd; The DOX+THIA group presented an improvement in EF and FS relative to the DOX group of rats; a 7-day thiamine administration induced a significant increase in antioxidant values SOD and GSH in heart tissue compared to its activity in DOX rats.	[82]
The study by Radonjic et al. focused on the effect of thiamine hydrochloride on the reversal of DOX-induced cardiotoxicity and compared it with the reversal in the absence of thiamine pre-treatment.	Cardiac contractility was significantly altered after DOX treatment and diminished by thiamine pre-treatment; Pre-treatment with thiamine hydrochloride before doxorubicin administration could decrease oxidative stress production, increase myocardial contractility, and enhance the antioxidant defense system.	[83]

TPP: thiamine pyrophosphate; NO: nitric oxide; ROS: reactive oxygen species; AGEs: advanced glycosylation end products; NF-κ B: nuclear factor kappa B; HUVECs: human umbilical cord isolated endothelial cells; B1: vitamin B1; IC50: half-maximal inhibitory concentration; HPLC: high-performance liquid chromatography; PDH: pyruvate dehydrogenase; DOX: doxorubicin; LVIDs: left ventricle internal dimension at end-systole; LVIDd: left ventricle internal dimension at end-diastole; LVPWs: left ventricular posterior wall thickness at end-systole; LVPWd: left ventricle internal dimension at end-diastole; THIA: thiamine; EF: ejection fraction; FS: fractional shortening; SOD: superoxide dismutase; GSH: glutathione.

## Abbreviations

AGEs	Advanced glycosylation end products
ATP	Adenosine triphosphate
B1	Vitamin B1
BAVA	Attenuated brachial artery vasoactivity
BBB	Blood–brain barrier
BCAAs	Branched-chain amino acids
CALR	Calreticulin
CF	Coronary flow
CI	Confidence interval
CPT1/2	Carnitine palmitoyltransferase 1 and 2
CVD	Cardiovascular disease
DLVP	Diastolic left ventricular development
DM	Diabetes mellitus
DOX	Doxorubicin
dp/dt max	Maximum rate of left ventricular development
dp/dt min	Minimum rate of left ventricular development
EAR	Estimated average requirement
EDV	Endothelium-dependent vasodilation
EF	Ejection fraction
eNOS	Endothelial nitric oxide synthase
ETK	Erythrocyte transketolase
ETKA	Erythrocyte transketolase activation assay
FAD	Flavin adenine dinucleotide
FADH2	Flavin adenine dinucleotide
FBS	Fasting blood sugar
FDA	Food and Drug Administration
FS	Fractional shortening
GDM	Gestational diabetes mellitus
GSH	Glutathione
HACL1	2-hydroxyacyl-CoA lyase 1
HbA1c	Glycated hemoglobin
HDLs	High-density lipoproteins
HF	Heart failure
HFpEF	Heart failure with preserved ejection fraction
HO•	Hydroxyl radicals
HO-1	Heme oxygenase 1
HOO•	Hydroperoxyl radicals
HPLC	High-performance liquid chromatography
HPLC–MS/MS	High-performance liquid chromatography–tandem mass spectrometry
HR	Heart rate
hs-CRP	High-sensitivity C-reactive protein
hTHTR-1	Human thiamine transporter-1
hTHTR-2	Human thiamine transporter-2
hTPPT	Human thiamine pyrophosphate transporter
HUVECs	Human umbilical vein endothelial cells
IC50	Half-maximal inhibitory concentration
ICAM-1	Intercellular adhesion molecule 1
IGT	Impaired glucose tolerance
iNOS	Nitric oxide inducible synthase
JAK2	Janus kinase 2
KGDHC	Alpha-ketoglutarate dehydrogenase complex
LDL	Low-density lipoprotein
LDLc	Low-density lipoprotein cholesterol
L-NAME	NG-nitro-L-arginine-methyl-ester
LV	Left ventricle
LVEF	Left ventricular ejection fraction
LVIDd	Left ventricle internal dimension at end-diastole
LVIDs	Left ventricle internal dimension at end-systole
LVPWd	Left ventricle internal dimension at end-diastole
LVPWs	Left ventricular posterior wall thickness at end-systole
MALDI-MS	Matrix-assisted laser desorption/ionization mass spectrometry
MDA	Malondialdehyde
MI	Myocardial infarction
MTPP-1	Mitochondrial thiamin pyrophosphate transporter 1
NADH	Nicotinamide adenine dinucleotide
NADPH	Nicotinamide adenine dinucleotide phosphate
NF-k B	Nuclear factor kappa B
NF-KB	Nuclear factor kappa-light-chain-enhancer of activated B cells
NIDDM	Non-insulin-dependent diabetes mellitus
NO	Nitric oxide
NOS	Nitric oxide synthase
OCT1	Organic cation transporter 1
OCT2/3	Organic cation transporter 2 and 3
OR	Odds ratio
P2Y6	Pyrimidinergic receptor P2Y6
PAD	Peripheral arterial disease
PARP	Poly (ADP-ribose) polymerase
PDH	Pyruvate dehydrogenase
PDK	Pyruvate dehydrogenase kinase
PPP	Pentose phosphate pathway
RBCs	Red blood cells
RBS	Random blood sugar
RDA	Recommended daily allowance
RF	Renal failure
ROS	Reactive oxygen species
SAM	Severe acute malnutrition
SLC19A1	Solute carrier family 19 member 1
SLC19A2	Solute carrier family 19 member 2
SLC19A3	Solute carrier family 19 member 3
SLC22A1	Solute carrier family 22 member 1
SLC25A19	Solute carrier family 25 member 19
SLC35F3	Solute carrier family 35 member F3
SLC44A4	Solute carrier family 44 member 4
SLVP	Systolic left ventricular development
SOD	Superoxide dismutase
Sp1	Transcription factor specificity protein-1
STIM1	Stromal Interaction Molecule 1
T1D	Type 1 diabetes
T2D	Type 2 diabetes
T2DM	Type 2 diabetes mellitus
TCA	Tricarboxylic citric acid
TD	Thiamine deficiency
TDDs	Thiamine deficiency disorders
ThDP	Thiamine diphosphate
THIA	Thiamine
THTR1	Thiamine transporter-1
THTR2	Thiamine transporter-2
TK	Transketolase enzyme
TMP	Thiamine monophosphate
TNF-α	Tumor necrosis factor-alpha
TPK1	Thiamine pyrophosphokinase 1
TPP	Thiamine pyrophosphate
TPPE	Effect of thiamine pyrophosphate
TPPT-1	Thiamine pyrophosphate transporter 1
TRMA	Thiamine-responsive megaloblastic anemia
TSH	Thyroid-stimulating hormone
US	United States
UTP	Uridine 5′-triphosphate
Vo2	Oxygen consumption
